# Supplementary material for: Decreased Systemic Monocyte Colony Protein-1 (MCP-1) Levels and Reduced sCD14 Levels in Curcumin-Treated Patients with Moderate Anxiety: A Pilot Study
Source: Antioxidants (Basel). 2024 Aug 29;13(9):1052. doi: 10.3390/antiox13091052 (PMC11429384; doi:10.3390/antiox13091052)
Supplement: Supplementary file 1 [file antioxidants-13-01052-s001.zip › antioxidants-3149999-supplementary.pdf]

Supplementary Materials

The diagram illustrates the enrolment of patients (n=81) and all study groups.

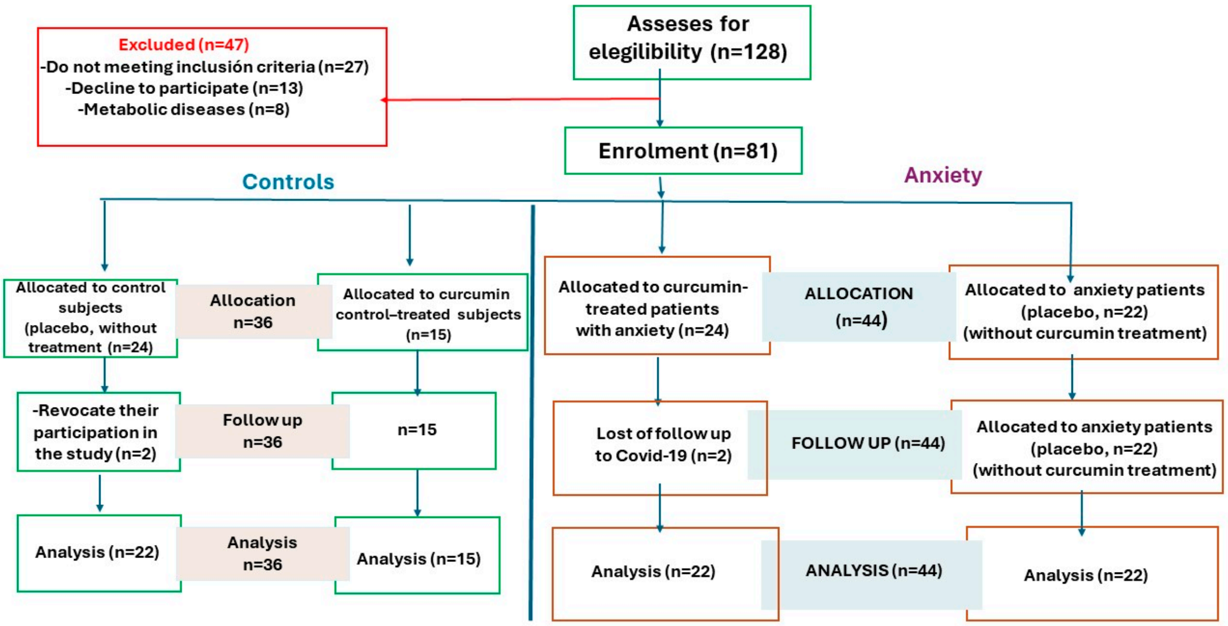

128 subjects filled up the Hamilton scale II and 81 of them were enrolled according their Hamilson scores. Patients with moderate anxiety were randomized (1:1) to receive curcumin (Meriva®) for 15 consecutive days (n=22) or placebo (Control subjects without anxiety, n=22). 44 have moderate anxiety and 47 subjects were excluded since they declined tehir participation or they do not meet the eligibility criteria.

In case of control subjects without anxiety, they were randomized to receive curcumin only for 15 days (1800 mg/day, twice, oral intake, n=15) or placebo (n=22).
